# Diffuse Alveolar Hemorrhage: An Unusual Presentation of Recurrent Malignant Melanoma

**DOI:** 10.7759/cureus.16324

**Published:** 2021-07-12

**Authors:** Patrick R McKillion, Sanjeev Shrestha, Andrew R Medvec, Amirahwaty Abdullah

**Affiliations:** 1 Internal Medicine, Geisinger Medical Center, Danville, USA; 2 Pulmonary and Critical Care Medicine, Geisinger Medical Center, Danville, USA

**Keywords:** diffuse alveolar hemorrhage, melanoma, bronchoscopy, bronchoalveolar lavage, malignancy

## Abstract

Diffuse alveolar hemorrhage (DAH) is a syndrome characterized by bleeding into the alveolar spaces of the lungs, secondary to disruption of the alveolar-capillary basement membrane. While numerous disease processes have been associated with DAH including certain malignancies, to the best of our knowledge, recurrent malignant melanoma has not been previously described in the literature as a cause of DAH. Here, we present a case of a 73-year-old female with a history of malignant melanoma of the left shoulder status post wide local incision two years prior, who presented with complaints of progressive shortness of breath without productive cough or hemoptysis. On examination, she was hypoxemic and required high-flow nasal cannula initiation. Initial investigation revealed a hemoglobin of 4.6 g/dL. Computed tomography of the chest with contrast revealed multiple areas of rounded infiltrates with air bronchograms, mediastinal adenopathy, and a right pleural effusion. Diagnostic bronchoscopy revealed findings of recent bleeding throughout the tracheobronchial tree with progressively bloody bronchoalveolar lavage (BAL) suggestive of DAH. BAL cytology was positive for malignant melanoma. After a comprehensive workup that excluded the common causes of DAH, we present the first case of DAH caused by recurrent malignant melanoma.

## Introduction

Diffuse alveolar hemorrhage (DAH) is a syndrome characterized by bleeding into the alveolar spaces of the lungs, secondary to disruption of the alveolar-capillary basement membrane. This disruption is most commonly a result of injury or inflammation of the arterioles, venules, or alveolar septal capillaries. Numerous disease processes have been associated with DAH, including systemic vasculitides, rheumatological diseases, drug-induced processes, and pulmonary infections. While certain malignancies have been implicated before, here we present the first reported case of DAH caused by undiagnosed recurrent malignant melanoma.

This article was previously presented as a poster presentation at the 2021 American Thoracic Society International Conference on May 14, 2021.

## Case presentation

**Figure 1:**
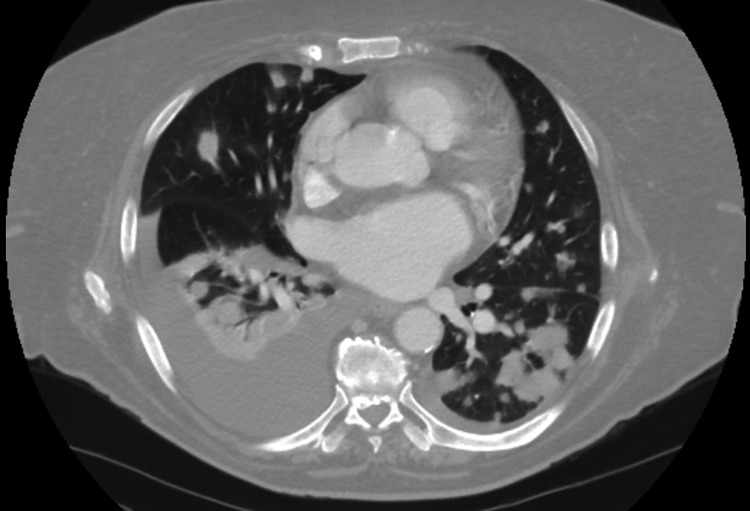
CT chest with contrast demonstrating multiple areas of rounded infiltrates with air bronchograms, mediastinal adenopathy, and right pleural effusion

A 73-year-old female with a history of hypertension, diabetes, and malignant melanoma of the left shoulder status post wide local incision two years prior, who presented with complaints of progressive shortness of breath without productive cough or hemoptysis. On examination, she was hypoxemic and required high-flow nasal cannula initiation. She appeared pale and had a normal S1 and S2 with diffuse crackles in all lung fields. Initial investigations revealed hemoglobin of 4.6 g/dL compared to her baseline of 13 g/dL the year prior. The admission chest radiograph demonstrated bilateral patchy nodular airspace opacities. Computed tomography of the chest with contrast revealed multiple areas of rounded infiltrates with air bronchograms, mediastinal adenopathy, and right pleural effusion (Figure 1).

**Figure 2:**
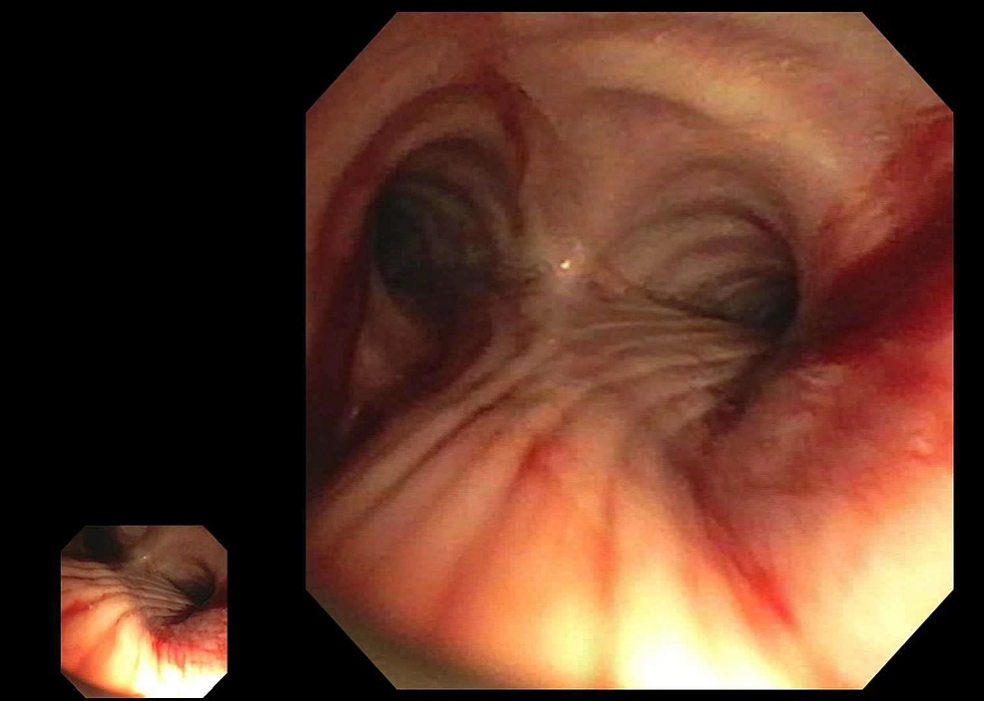
Bronchoscopy with recent bleeding throughout the tracheobronchial tree

Due to concerns for lung metastasis, she underwent diagnostic bronchoscopy, which revealed findings of recent bleeding throughout the tracheobronchial tree prior to any instrumentation (Figure 2).

**Figure 3:**
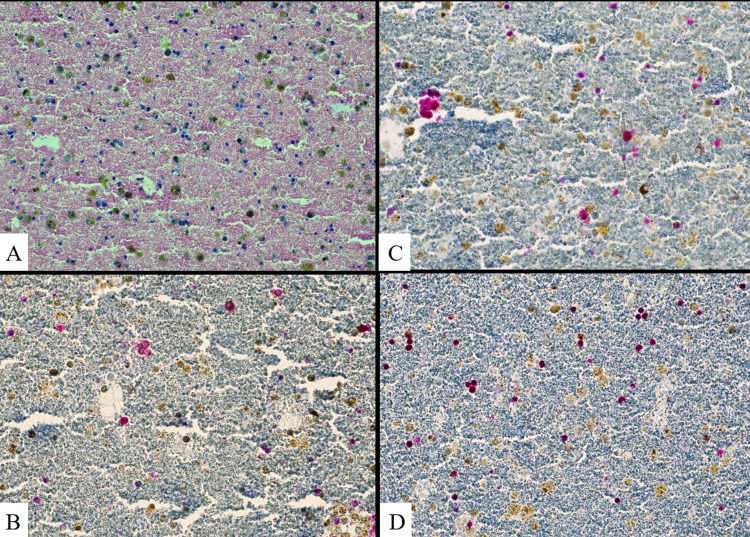
Cell block preparation showing melanoma cells on (A) H and E staining, (B) immunostaining for MART-1, (C) immunostaining for S100, and (D) immunostaining for SOX10 MART-1: melanoma antigen recognized by T-cells; SOX10: sex-determining region Y-box transcription factor 10

The bronchoalveolar lavage (BAL) was progressively bloody suggestive of DAH. Autoimmune disease evaluation, including antinuclear antibody (ANA), antineutrophil cytoplasmic antibodies (ANCA) battery, rheumatoid factor (RF), cyclic citrullinated peptide (CCP), anti-glomerular basement membrane antibodies (anti-GBM ab), anti-beta 2 glycoprotein ab, and anti-cardiolipin ab were all found to be non-diagnostic. Blood and BAL cultures did not show evidence of an active infection. BAL cytology was positive for malignant melanoma (Figures 3A-3D).

**Figure 4:**
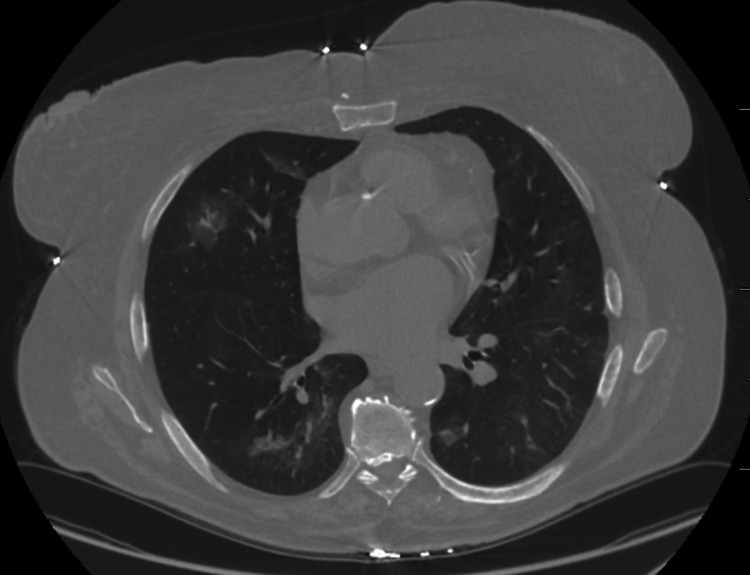
Follow-up CT scan with improvement of previously seen infiltrates after initiation of chemotherapy

Oncology evaluated with plans for palliative chemotherapy, and the patient has been on dabrafenib and trametinib with good response (Figure 4).

## Discussion

This case is unique because our patient’s initial presentation with hypoxic respiratory failure secondary to DAH unveiled metastatic melanoma. To the best of our knowledge, this presentation has not been previously described in the literature. Our initial workup was to rule out more common causes of DAH like vasculitis. After extensive testing, common etiologies were excluded. Interestingly, the cytology revealed a heterogeneous B-Raf proto-oncogene (BRAF) V600E and BRAF V600K population of metastatic melanoma even though prior excisional margins were thought to be clean. Per the literature review, melanoma reoccurrence with distant metastasis from the primary site following complete surgical resection is fairly common as it is seen in approximately 30% of patients [1], with preferential spread to the thorax [2]. Also, melanoma metastases have been associated with a higher risk of hemorrhage; for instance, melanoma metastases are the most common cause of hemorrhagic metastasis in the brain [3].

When treating cancer patients, it has been a well-described balancing act between risk of hemorrhage and prevention of thrombosis. During our patient’s admission, she was newly diagnosed with atrial fibrillation which could not be anticoagulated while inpatient due to DAH leading to a higher risk of stroke. Also of interest, our patient was started on dabrafenib and trametinib which have been shown in a few case reports to cause hemorrhagic conversion of metastasis thought secondary to rapid necrosis [4,5]. Our patient has had an excellent response to her dual therapy of dabrafenib and trametinib based on a nine-month follow-up positron emission tomography and computed tomography (PET-CT) and has experienced no further episodes of hemorrhage with a stable hemoglobin.

## Conclusions

While numerous disease processes have been associated with DAH including certain malignancies, to the best of our knowledge metastatic melanoma has not been previously described in the literature as a cause of DAH. This case report was written in hopes to shed light on broadening the differential to include metastatic melanoma in a patient presenting with DAH with remote history of melanoma.
